# Sideroblastic anemia: functional study of two novel missense mutations in *ALAS2*

**DOI:** 10.1002/mgg3.202

**Published:** 2016-01-13

**Authors:** Manuel Méndez, María‐Isabel Moreno‐Carralero, Marta Morado‐Arias, María‐Cristina Fernández‐Jiménez, Silvia de la Iglesia Iñigo, María‐José Morán‐Jiménez

**Affiliations:** ^1^ Instituto de InvestigaciónHospital 12 de OctubreMadridSpain; ^2^ Departamento de HematologíaHospital La PazMadridSpain; ^3^ Departamento de HematologíaComplejo Hospitalario de ToledoToledoSpain; ^4^ Departamento de HematologíaHospital Doctor NegrínLas Palmas de Gran CanariaMadridSpain

**Keywords:** ALAS2, in silico analysis, prokaryotic expression, sideroblastic anemia

## Abstract

**Background:**

X‐linked sideroblastic anemia (XLSA) is a disorder characterized by decreased heme synthesis and mitochondrial iron overload with ringed sideroblasts in bone marrow. XLSA is caused by mutations in the erythroid‐specific gene coding 5‐aminolevulinate synthase (*ALAS2*). Anemia in XLSA is extremely variable, characteristically microcytic and hypochromic with poikilocytosis, and the red blood cell distribution width is increased and prominent dimorphism of the red cell population. Anemia in XLSA patients responds variably to supplementation with pyridoxine.

**Methods and Results:**

We report four patients with XLSA and three mutations in *ALAS2*: c.611G>A (p.Arg204Gln), c.1218G>T (p.Leu406Phe) and c.1499A>G (p.Tyr500Cys). The in silico predictions of three ALAS2 mutations and the functional consequences of two ALAS2 mutations were assessed. We performed in silico analysis of these mutations using ten different softwares, and all of them predicted that the p.Tyr500Cys mutation was deleterious. The in vitro prokaryotic expression showed that the p.Leu406Phe and p.Tyr500Cys mutations reduced the ALAS2 specific activity (SA) to 14% and 7% of the control value, respectively.

**Conclusion:**

In view of the results obtained in this study, a clear relationship between genotype and phenotype cannot be established; clinical variability or severity of anemia may be influenced by allelic variants in other genes or transcription factors and environmental conditions.

## Introduction

X‐linked sideroblastic anemia (XLSA), OMIM: 300751, is the most common disorder of a group characterized by decreased heme synthesis and mitochondrial iron overload with ringed sideroblasts in the bone marrow (Camaschella 2009; Harigae and Furuyama 2010; Fujiwara and Harigae 2013). XLSA is caused by mutations in the specific erythroid gene coding 5‐aminolevulinate synthase (*ALAS2*) localized on chromosome Xp11.21. ALAS (EC 2.3.1.37) is the first enzyme of heme biosynthesis and catalyzes the condensation of glycine and succinyl coenzyme A into 5‐aminolevulinic acid (ALA) in the mitochondria.

Anemia in XLSA is extremely variable and characteristically microcytic and hypochromic with poikilocytosis, and the red blood cell distribution width is usually increased. Prominent dimorphism (micro‐ and normo‐ or even macrocytic) of the red cell population has been noted, especially in females with inactivation of the normal X chromosome (Camaschella 2009). Anemia in many XLSA patients responds variably to supplementation with pyridoxine (vitamin B6) which is converted into pyridoxal 5‐phosphate (PLP), the active cofactor essential for ALAS2 activity.

Most of the ninety *ALAS2* mutations reported so far, which are responsible for XLSA, are missense, and some of them have been expressed in *Escherichia coli* to study the activity of mutated protein (Cotter et al. 1992, 1994, 1995; Cox et al. 1994; Prades et al. 1995; Furuyama et al. 1997, 1998, 2003, 2006; Harigae et al. 1999a,b; Ducamp et al. 2011; Bishop et al. 2012).

Here, we report four patients with XLSA and three different mutations in *ALAS2*. Two siblings had a mutation that had already been described, and two patients had novel mutations, one of them has been recently published by some authors of this work. We performed the in silico analysis of mutations and the in vitro study to assess functional consequences of the two novel cases.

## Patients and Methods

### Ethical compliance

The written informed consents were obtained according to the local guidelines for genetic studies, and were in accordance with the ethical guidelines of the Declaration of Helsinki for Human Research.

### Patients

#### Patient 1

A male in his mid‐thirties was studied because of severe hypochromic, microcytic anemia, and a high‐ferritin level (Table 1). Bone marrow aspiration presented 41% of ringed sideroblasts at diagnosis (Table 1). The diagnosis of congenital sideroblastic anemia (CSA) was set and pyridoxine treatment (300 mg/day) was started normalizing the hemoglobin (Hb) level. At 51, he was diagnosed with monoclonal gammopathy of undetermined significance (MGUS), Hb level stayed within the normal range and no sideroblasts in the bone marrow were observed.

**Table 1:** Hematological and biochemical parameters of patients with sideroblastic anemia

	R.S. (%)	Hb (g/dL)	Hematocrit (%)	MCV (fL)	MCH (pg)	CHCM (g/dL)	RDW (%)	Platelet (×10^3^/*μ*L)	Serum iron (*μ*/g per dL)	Tf (mg/dL)	TIBC (*μ*g/dL)	Tf sat. %	Ferritin (ng/mL)	Hepcidin (ng/mL)
Case 1	41	5.0	15.0	65.1	19.7	30.2	28.7	383	287	232	294	98	632	
M	0	13.8	39.8	77.9	27.0	34	11.9	269	87	326	414	21	40	20
Case 2	11	11.3	37.2	73.2	22.2	30.4	21.2	326	113	300	381	30	878	
M	0	14.7	43.0	87.0	29.8	34.2	12.6	258	93	288	365	25	110	3
Case 3	27	9.2	27.5	77.5	27.1	33.4	37.0	409	309	261	332	93	393	2
F		12.3	37.2	75.0	25.0	33.0	18.6	394	122	279	354	36	212	12
Case 4	No data													
M	10	11.1	37.9	73.4	21.6	29.4	20.0	64	179	140	178	101	3374	42

Data for each patient at diagnosis in the first row and at present in the second row are shown. M, male; F, female; R.S., Ringed sideroblasts. Normal values: Hemoglobin, Hb: 13.0–17.3 (females 12.0–15.0) (anemia: females <12, males <13); Hematocrit: 38.9–51.4 (females 37.0–47.0); Mean Corpuscular Volume, MCV.: 80.0–97.0 (microcytosis<80); medium corpuscular hemoglobin**,** MCH: 26.0–33.0; concentration medium corpuscular hemoglobin, CHCM: 31.2–36.0; red cell distribution width, RDW: 11.6–14.5; platelet: 125–350; serum iron: 70–180, transferrin, Tf: 200–360; total iron binding capacity, TIBC: 291–430; transferrin saturation, Tf sat.: 15–50, ferritin: females 20–150, males 30–300; hepcidin: 2–20.

#### Patient 2

A male in his mid‐thirties, brother of patient 1, had mild microcytic anemia with two populations of red cells, and 11% of ringed sideroblasts in the bone marrow aspirate (Table 1). He was also studied and diagnosed with CSA and was treated with pyridoxine (300 mg/day).

Currently patients 1 and 2 are in their fifties, and have hematological parameters within the normal range and no profile of iron overload (Table 1).

#### Patient 3

We have previously published this patient (Rollon et al. 2015). Briefly, a female in her late‐thirties was diagnosed with anemia that became more severe from the onset of pregnancy. Analytical and morphological studies revealed a mild hypochromic, microcytic anemia, abnormalities in red blood cell series, and 27% ringed sideroblasts in the bone marrow (Table 1). The iron profile parameters were indicative of iron overload, and a moderate iron deposit in the liver was observed by abdominal magnetic resonance imaging (MRI). The patient responded partially to a low dose of pyridoxine during pregnancy and reached a normal Hb level with 60 mg/day after delivery (Rollon et al. 2015). The red cell abnormalities improved, but did not disappear, despite the restoration of Hb levels, 2 years later (second row on Table 1).

#### Patient 4

**Figure 1:**
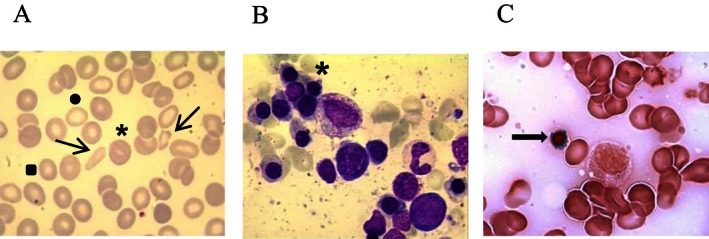
(A) Peripheral blood smear of patient 4 showing anisopoikilocytosis (arrows), microcytosis (square), and hypochromia (circle) with basophilic stippling (asterisk). (B and C) Bone marrow smears of patient 4 showing erythroid hyperplasia (asterisk) without dysplasia and ringed sideroblasts (arrow).

A male, now in his late forties, was diagnosed with CSA at the end of his second decade when required a blood transfusion after a traumatic accident. Anemia was detected and bone marrow smear morphology was compatible with the diagnosis of sideroblastic anemia. He has not received a blood transfusion since then. There was no information about hematological parameters or morphological details at that time. He has been treated with B6 vitamin (pyridoxine 300 mg/day) and folic acid (5 mg/day) for 30 years from the initial diagnosis to this day. Whenever the vitamin treatment was interrupted, anemia reappeared (minimal Hb level 7.8 g/dL). Hemostasis and coagulation studies revealed thrombocytopenia due to chronic hepathopathy secondary to hepatitis infection (HCV and HCB) with portal hypertension. Morphology of a blood smear showed anisocytosis, microcytosis and hypochromia with basophilic stippling (Fig. 1). Analytical hematology disclosed altered red blood cell series. Bone marrow aspiration shows erythroid hyperplasia with no dysplastic features and bone marrow iron storages increased to 75% of type I or II and 10% of ringed sideroblasts (Fig. 1, Table 1, current data). The iron profile parameters were elevated and indicative of iron overload, probably due to chronic dyserythropoiesis. Abdominal MRI showed severe hepatic iron overload, 360 *μ*mol Fe/g tissue (normal <36 *μ*mol Fe/g tissue) measured following University of Rennes protocol), and subsequently iron chelation therapy was established in 2014, 31 years after diagnosis, using high doses of deferasirox (30 mg/kg body weight/day), with slow response. Treatment of chronic hepatitis was initiated with sofosbuvir (400 mg/day), and he is much improved. To exclude other causes or iron overload, we have studied the genes: *HFE*,* HJV*,* HAMP*,* TFR2*, and *SLC40A1*; the GenBank accession numbers: NG_008720.2, NG_011568.1, NG_011563.1, NM_003227.3, and NG_009027.1, respectively.

### Molecular analysis of *ALAS2* gene

Genomic DNA was extracted from a peripheral blood sample. Polymerase chain reactions (PCR) were performed to amplify the coding sequences, exon‐intron junctions, and 5′ and 3′ untranslated regions using specific oligonucleotides. PCR products were sequenced bidirectionally with the BigDye terminator v1.1 cycle sequencing kit (Applied Biosystems, Austin, TX, USA) on the ABI Prism 3130x1 Genetic Analyzer (Applied Biosystems, Foster city, CA, USA). Sequences were reviewed and compared with the reference sequence GenBank accession number NG_008983.1 by ClustalW2 software http://www.ebi.ac.uk/Tools/msa/clustalw2/. Mutations were confirmed by sequencing two different PCR products. The novel mutations were searched in 50 control individuals.

### In silico analysis of ALAS2 mutations

Computational analyses were used to predict the effect of the missense substitutions found in the study (p.Arg204Gln, p.Leu406Phe, and p.Tyr500Cys) on the ALAS function. In silico methods were based on protein multiple sequence alignments of the gene of interest across multiple species and on crystal structure, amino acids located at enzymatic active sites or at binding sites, amino acids with low or high solvent accessibility, amino acid located in alpha helix. We used the sequence with the accession number P22557 for ALAS2 of UniProtKB database. The in silico programs used are: BLOSUM62 (http://www.uky.edu/Classes/BIO/520/BIO520WWW/blosum62.htm), Grantham (https://gist.github.com/arq5x/5408712/raw/be166b658d6c332ba0b0c357612026ccf7f60d11/grantham-dict.py), SIFT (http://sift.bii.a-star.edu.sg/), SNP3D (http://www.snps3d.org/), Panther (http://www.pantherdb.org/), Provean (http://provean.jcvi.org/index.php), Mutation assessor (http://mutationassessor.org/), I‐Mutant Suite (http://gpcr2.biocomp.unibo.it/cgi/predictors/I-Mutant3.0/I-Mutant3.0.cgi), GVGD (http://agvgd.iarc.fr/cgi-bin/agvgd_output.cgi), Polyphen 2.0 (http://genetics.bwh.harvard.edu/pph2/).

### Normal and mutants *ALAS2* expression constructs

First, RNA was extracted from peripheral blood of a healthy individual and reverse transcription was performed to obtain cDNA, then PCR was done using ALAS2 sense and antisense oligonucleotides (Table 2) to generate normal *ALAS2* cDNA. This PCR product was digested with *EcoRI* and *HindIII* restriction enzymes, and ligated into the *EcoRI* and *HindIII* linearized and dephosphorylated pKK223‐3 vector (Pharmacia Biotechnology, Piscataway, NJ, USA). *Escherichia coli* JM109 competent cells (Promega Corporation, Madison, WI, USA) were transformed with this pKK‐ALAS2 construct, and cultured overnight at 37°C and 250 rpm in LB medium with 100 *μ*g/mL ampicillin, then plasmid DNA was extracted and coding *ALAS2* sequence was verified by sequencing.

**Table 2:** Oligonucleotides used to construct wild type and mutants *ALAS2* cDNA. Oligonucleotides (5′→3′)

ALAS2 sense	*GGGCC* *GAATTC* **ATG**CAAATCCACCTTAAGGCAAC
ALAS2 internal sense	CATCTGTCCCCTCGAGGAGTTGTG
ALAS2‐L406F sense	CACCCGTGACTT**T**GTGGACATGGTG
ALAS2‐L406F antisense	CACCATGTCCAC**A**AAGTCACGGGTG
ALAS2‐Y500C sense	GCATGGCATCT**G**TGTGCAGGCCATC
ALAS2‐Y500C antisense	GATGGCCTGCA**C**ACAGATGCCATGC
ALAS2 antisense	*CCCGG* *AAGCTT* **TCA**GGCATAGGTGGTGAC

The ALAS2 sense and antisense oligonucleotides show the original sequence of *ALAS2* and added bases in italic; the *EcoRI* and *HindIII* restriction sites are underlined in sense and antisense oligonucleotides, respectively. The initiation codon (ATG) and the stop codon (TCA, reverse complement) of *ALAS2* cDNA are in bold in ALAS2 sense and antisense oligonucleotides, respectively. The ALAS2 internal oligonucleotide shows the *XhoI* restriction site underlined. The mutations introduced are in bold and underlined in the mutagenesis oligonucleotides.

The c.1218G>T (p.Leu406Phe) and c.1499A>G (p.Tyr500Cys) mutations were introduced into the pKK‐ALAS2 construct by site‐directed mutagenesis in two PCR steps using the ALAS2 oligonucleotides shown in Table 2 as follows: the ALAS2 internal sense and ALAS2‐L406F (or ALAS2‐Y500C) antisense oligonucleotides ,and the ALAS2‐L406F (or ALAS2‐Y500C) sense and ALAS2 antisense oligonucleotides were used in the first two PCRs; these two PCR products were used as templates in a second PCR using ALAS2 internal sense and ALAS2 antisense oligonucleotides. Then, these two PCRs products were purified and digested with the *XhoI* and *HindIII* enzymes, and ligated into *XhoI* and *HindIII* digested and purified pKK‐ALAS2 construct to generate pKK‐ALAS2‐L406F and pKK‐ALAS2‐Y500C constructs. *Escherichia coli* cells were transformed with these two mutant constructs, cultured overnight, and then plasmids were extracted. The coding ALAS2 regions of the pKK‐ALAS2‐L406F or pKK‐ALAS2‐Y500C constructs were verified by sequencing. Stocks with glycerol of each culture, pKK‐ALAS2, pKK‐ALAS2‐L406F, pKK‐ALAS2‐Y500C, and pKK223‐3, were stored at −80°C.

### In vitro expression of normal and mutants ALAS2

An aliquot of each stock was grown overnight in LB‐ampicillin; the next day an aliquot of each culture was grown in 10 mL LB‐ampicillin to logarithmic phase, and induced by adding isopropyl b‐d‐thiogalactoside to the final concentration of 5 mmol/L for 3 h. Cultures were then centrifuged at 10,000*g* for 10 min at 4°C and pellets were kept at −80°C until the ALAS enzymatic reaction was performed. Pellets were sonicated on ice in 200 *μ*L HEPES 50 mmol/L: pH 7.5, 3 times for 15 sec at an intensity of 7 watts in a sonifier liquid processor, 150D (Branson Ultrasonic corporation, Danbury, CT, USA). The total protein concentration was quantified by Bio‐Rad DC protein assay (Bio‐Rad Laboratories, Hercules, CA, USA).

ALAS activity was performed with 100 *μ*g of proteins and 50 *μ*L of a mixture containing the substrates, cofactor and an inhibitor of the next enzyme of the heme synthetic pathway (1 mmol/L succinyl‐CoA, 700 mmol/L glycine, 0.5 mmol/L PLP, and 0.07 mmol/L 4,6‐dioxoheptanoic acid or succinyl acetone, respectively) in a final reaction volume of 300 *μ*L of HEPES 50 mmol/L pH 7.5. The reaction was stopped by adding 40 *μ*L trichloroacetic acid 50% one immediately before incubation (blank, T0) and the other after incubation at 37°C for 30 min in a shaking water bath (duplicate samples, T30). Samples were centrifuged at 20,000*g* for 10 min at 4°C and the supernatants were collected. Condensation of two molecules of ALA to one molecule of pyrrole was produced by heating at 95°C for 10 min in 250 *μ*L supernatant with 250 *μ*L sodium acetate 1 mol/L pH 4.6 and 50 *μ*L acetylacetone; then cooled and 500 *μ*L of modified Ehrlich's reagent was added (Mauzerall and Granick 1956). The absorbance at 553 nm was measured at 10 min, absorbance at T0 correspond to endogenous pyrroles present in the bacterial lysate and is substracted to absorbance at T30. Concentrations of ALA were calculated using *ε*_mM_ = 58 for the Ehrlich pyrrole color. The SA was expressed in nmol ALA/mg protein per hour.

### HUMARA assay

We performed the human androgen receptor gene polymorphism assay (HUMARA) on the DNA from patient 3 to assess the X chromosome inactivation. The assay was carried out according to that described by Jones (2014) DNA was digested with *HpaII*, a methylation‐sensitive enzyme, then amplified with a labeled‐forward oligonucleotide‐FAM and a unlabeled‐reverse oligonucleotide for the androgen receptor locus. The PCR products were separated by capillary electrophoresis using the fluorescein channel with analysis performed on GeneMapper Generic Module using the MLPA Analysis method (Genetic Analyzer 3130; Applied Biosystems Inc.).

## Results and Discussion

**Figure 2:**
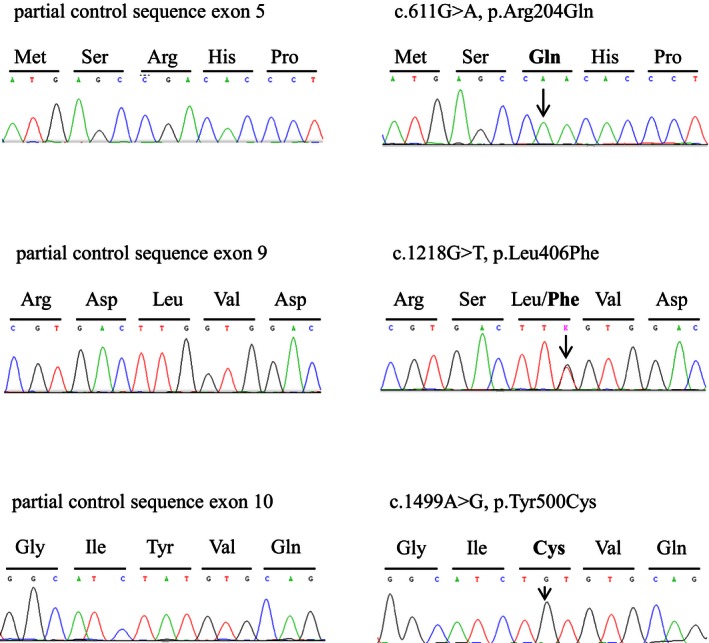
Electropherograms show the mutations identified in this study: control sequences (left panels) and mutated sequences (right panels) of 5‐aminolevulinate synthase *ALAS2* gene (NG_008983.1).

We found three missense mutations in the *ALAS2* gene: c.611G>A in exon 5 (p.Arg204Gln) in hemizygosis in two brothers (patients 1 and 2), this mutation had already been reported (Harigae et al. 1999b; Harigae and Furuyama 2010) c.1218G>T in exon 9 (p.Leu406Phe) in heterozygosis (patient 3) (Rollon et al. 2015), c.1499A>G in exon 10 (p.Tyr500Cys) in hemizygosis (patient 4) not previously published (Fig. 2). The novel mutations were absent in 50 control individuals.

We used ten different computer programs to analyze the functional impact of the mutations: p.Arg204Gln, p.Leu406Phe, and p.Tyr500Cys mutations were predicted to be deleterious in two, five, and all of the softwares, respectively (Table 3).

**Table 3:** In silico analysis of ALAS2 mutations

ALAS2 (P22557)	BLOSUM62	Grantham	SIFT	SNPS3	PANTHER	PROVEAN	Mutation Assessor	I‐mutant suite	GVGD	Polyphen
Arg204Gln	Common 1	Conservative 43	Tolerated	Nondeleterious 2.35	Neutral/deleterious −1.96	Deleterious/neutral −2.504	Functional impact low 1.165	Decrease stability −0.70	Moderate deleterious C35	Possibly damaging 0.725
Leu406Phe	Common 0	Conservative 22	Tolerated	Nondeleterious 0.34	Possible deleterious −2.89	Deleterious −3.715	Functional impact Medium 2.815	Large decrease stability −1.37	Weak deleterious C15	Probably damaging 1
Tyr500Cys	Rare ‐2	Radical 194	Not tolerated	Deleterious −2.99	Most likely deleterious −5.72	Deleterious −8.688	Functional impact High 4.08	Large decrease stability −1.10	Most likely deleterious C65	Probably damaging 1

Predictions for missense substitution and score are indicated for every software analysis. BLOSUM62: score range from −4 to −1 for rare, from 0 to 3 for common substitutions and from 4 to 11 for identities. The cut‐off of 3.5 is the predictive value to classify deleterious *versus* neutral substitution, Grantham: score from 5 to 215: <50 conservative, 51–100 moderately conservative, 101–150 moderately radical, >151 radical substitutions, SIFT: score >0.05 indicative of tolerated substitution, score ≤0.05 indicative of not tolerated or deleterious substitution, SNP3D: score from positive to negative: from nondeleterious to deleterious substitution. The larger the score the more confident the classification. Accuracy is significantly higher for scores greater 0.5 or less than −0.5, PANTHER: score from 0 to −10, from neutral to most likely to be deleterious, a cutoff of −3 corresponds to a 50% probability that is deleterious, PROVEAN: the default score threshold is currently set at −2.5 (score < −2.5 deleterious, score > −2.5 neutral), Mutation Assessor: score from −5.76 to 5.37, score <1.938 is polymorphism associated and score >1.938 is disease associated, I‐Mutant Suite: DDG value prediction: DDG <−0.5 large decreased stability, DDG>0.5 large increased stability, −0.5 ≤ DDG ≤0.5 neutral stability, GVGD: prediction ordered grades: C0, C15, C25, C35, C45, C55, C65, from less likely (C0) to most likely (C65) deleterious substitution to interfere with function, Polyphen2.0: score from 0 to 0.14 benign, from 0.15 to 0.84 possible damaging, and from 0.85 to 1 probably damaging substitutions.

The p.Leu406Phe and p.Tyr500Cys mutations reduced the ALAS2 SA to 14% and 7% of the control value, respectively (Table 4).

**Table 4:** Prokaryotic expression of *ALAS2* missense mutations

	ALAS2‐specific activity nmol ALA/mg total protein/h	Residual activity %
pKK223‐3	7.16 ± 5.05 (0–12.93)	0
pKK‐ALAS2	45.21 ± 13.27 (30.40–61.85)	100
pKK‐ALAS2‐L406F	12.49 ± 6.29 (5.24–25.16)	14
pKK‐ALAS2‐Y500C	9.82 ± 3.49 (5.42–14.68)	7

ALAS2‐Specific activity (SA) was determined in 6–9 independent experiments. Values are expressed as mean ± standard deviation (range). Residual activity was calculated as 100 × (SA‐SA_pkk223‐3_)/(SA_pkk‐ALAS2_‐SA_pkk223‐3_).

The ALAS2 is the first enzyme of the heme synthesis in erythroid cells, it requires PLP as a cofactor to display its activity, and regulates the supply of protoporphyrin IX (PPIX) in the final step of the pathway. This last step is catalyzed by the ferrochelatase which introduces reduced iron (Fe^2+^) into the PPIX. The ALAS2 deficiency causes a deficit in the heme formation and an accumulation of the unused iron in mitochondria of erythroblasts. More than half of patients respond to oral pyridoxine treatment, leading to restoration of the enzymatic activity and improvement of the hypochromic and microcytic anemia (Camaschella 2009; Harigae and Furuyama 2010; Fujiwara and Harigae 2013). Pyridoxine responsiveness is considered: (a) complete when anemia is cured or nearly cured, Hb level ≥11 g/dL; (b) partial when anemia is improved, Hb level increases by ≤1 g/dL or symptoms are alleviated; or (c) absent when Hb level does not increase or increases but ≤1 g/dL (Liu et al. 2013). The phenotype of XLSA is variable concerning abnormalities in red blood cells, degree of microcytosis and anemia, iron overload, and age at diagnosis (Ducamp et al. 2011; Liu et al. 2013; Campagna et al. 2014; Kaneko et al. 2014). Hemizygous males affected with XLSA are diagnosed in the first two decades of life with microcytic anemia of variable degrees, or in middle age with manifestations of iron overload. Most of the heterozygous females have no clinical signs of anemia because the normal ALAS2 protein sustains the production of the necessary amounts of red blood cells, but the skewed X‐inactivation because of congenital, acquired or age‐dependent leads to XLSA (Busque et al. 1996; Cazzola et al. 2000; Aivado et al. 2006; Sankaran et al. 2015).

Patients 1 and 2 are brothers, diagnosed with CSA in their thirties, who have shown a complete response to pyridoxine treatment. Patient 3, a female**,** treated occasionally with iron because of anemia and diagnosed with XLSA in the fourth decade of life when anemia worsened because of pregnancy (Rollon et al. 2015). This condition would have induced highly skewed X‐inactivation (98:2), as observed by HUMARA assay. She responded well to a lower dose of pyridoxine than that of patients 1, 2, and 4. Patient 4, a male diagnosed with CSA in the second decade of life when a blood transfusion was required due to an accident. He responded to pyridoxine treatment with regard to hemoglobin level (increasing from 7.8 to 11.1 g/dL) but red cell abnormalities did not disappear. Moreover, he has a more severe hepatic iron overload than the others, probably triggered by chronic dyserythropoiesis and aggravated by hepatopathy secondary to hepatitis infection. Other genetic causes of iron overload were discarded as we only found the c.845G>A (p.Cys282Tyr) mutation in heterozygosis in *HFE* gene, as this mutation is not considered as pathogenic in heterozygous state (EASL 2010).

The clinical phenotype of these patients is different, age of diagnosis, hematological and biochemical parameters, responsiveness to pyridoxine, even in patients with the same mutation such as patients 1 and 2. Clinical response to B6 depends on the site of the mutation, affecting or not the binding region pyridoxine, dose B6, and iron overload status. Sometimes patients not respond until chelating therapy is implemented because iron in the mitochondria interferes with the function of the enzyme through iron responsive proteins (Donker et al. 2014). Although we have not studied other phenotype modulators, we speculate that clinical variability or severity of anemia may be influenced by allelic variants in other genes, as reported for other hematological disorders (Steinberg and Sebastiani 2012; Pepe et al. 2014; Danjou et al. 2015; Sorolla et al. 2015; Vicari et al. 2015).

The p.Arg204Gln mutation was reported to have a residual activity of 35% (Harigae et al. 1999b). This study shows that the two novel mutations p.Leu406Phe and p.Tyr500Cys in ALAS2 result in a decreased residual activity in vitro, 14% and 7%, respectively (Table 4). Moreover, two, five, and ten of the ten in silico analysis used predicted that the p.Arg204Gln, p.Leu406Phe, and p.Tyr500Cys mutations, respectively, were deleterious. When more in silico programs predict that the mutation has a negative impact on the functionality or stability of the ALAS2 (Table 3), the lower the residual activity.

To date, 90 mutations have been reported in the *ALAS2* gene causing XLSA: 76 missense/nonsense mutations, 3 splicing mutations, 5 small deletions, 1 small indel, 3 gross deletions (http://www.hgmd.cf.ac.uk/ac/all.php). These mutations are located from exon 4 to exon 11, spanning exon 9 containing Lys391 where PLP is covalently bound. The missense mutations could alter the conformation of ALAS2, and thus its affinity for PLP, would be responsible for pyridoxine responsiveness in patients. The nonsense mutations that create a premature stop codon and those mutations that destabilize the ALAS2 protein would be responsible for pyridoxine unresponsiveness in patients. The regulatory mutations in the promoter lead to the impairment of transcriptional regulation of *ALAS2* gene would be responsible for pyridoxine‐refractory patients (Bekri et al. 2003; Barton et al. 2005; Lee et al. 2009; Campagna et al. 2014; Kaneko et al. 2014).

Although no crystal structure of human ALAS2 is available, the high degree of conservation between human ALAS2 and *Rhodobacter capsulatus* (*Rc*) ALAS, particularly in the active site, makes the crystallographic structure of ALAS *Rc* a model to locate mutations in the structure and to elucidate their structural and functional implications on XLSA (Astner et al. 2005).

The residue Arg204 (Gln61 in *Rc* homolog), located on the surface of ALAS2, at a loop between the N‐terminal domain and the catalytic domain; the Arg204 forms a salt bridge to Asp173 (Gly31 in *Rc* homolog), the Arg204Gln substitution would disrupt this interaction affecting a glycine‐rich region of the second ALAS2 monomer situated below, and weakening the interaction between the N‐terminal domain and the catalytic domain (Astner et al. 2005). The residue Leu406 (Met263 in *Rc* homolog), located on the protein surface, at the *α*9‐helix of the catalytic domain. In the *Rc* homolog, the *α*9‐helix contacts with a loop between *α*1‐helix and *α*2‐helix in the N‐terminal of the second ALAS monomer (http://swift.cmbi.ru.nl/servers/html/index.html). The mutation Leu406Phe leads to the replacement of an aliphatic Leu by an aromatic Phe possibly destabilizing the interaction and homodimer conformation. The residue Tyr500 (Tyr357 in *Rc* homolog), located at the *β*12‐sheet in the C‐terminal domain between two loops (*β*11‐sheet and *α*14‐helix, and *α*14‐helix and *β*12‐sheet) involved in the open and closed state conformations of the active‐site channel. In ALAS *Rc* homolog, Tyr357 contacts with Ser87 of the second ALAS monomer, in a glycine‐rich stretch of the catalytic domain involved in substrate binding (http://swift.cmbi.ru.nl/servers/html/index.html). In human ALAS2, these amino acid residues are conserved (Tyr500 and Ser230). The mutation Tyr500Cys leads to the replacement of an aromatic residue Tyr to a significantly shorter and uncharged Cys with a sulfhydryl that probably interferes in the conformation or stability of the active site, or in the glycine access and binding to ALAS2.

The in silico and in vitro studies showed that p.Tyr500Cys mutation had worse consequences on protein function that the other mutations analyzed, and the phenotype of patient 4 was more severe than that of the other patients. Although we have no data at diagnosis of this patient, pyridoxine treatment ameliorated symptoms of anemia through his life but clinical data remain outside the normal range. The in silico and in vitro studies showed that p.Leu406Phe mutation had an intermediate deleterious effect on ALAS2 function compared with the other mutations analyzed. Nonetheless, patient 3 is a female carrying this mutation and clinical signs might be compensated because of functionality of normal ALAS2. The anemia could possibly be aggravated because of skewed X‐inactivation at pregnancy.

No significant correlations between mutations in the *ALAS2* gene and severity of anemia in XLSA patients have been reported so far. In view of the results obtained in this work, a clear relationship between genotype and phenotype cannot be established; clinical variability or severity of anemia may be influenced by allelic variants in other genes or transcription factors and, environmental conditions.

## Conflict of Interest

The authors declare no conflict of interest.
